# Systematic review and meta-analysis on the effect of olive oil in the treatment of periodontal diseases

**DOI:** 10.3389/froh.2025.1735845

**Published:** 2025-12-18

**Authors:** Nansi López-Valverde, Antonio López-Valverde, José A. Blanco Rueda

**Affiliations:** 1 Department of Surgery, University of Salamanca, Salamanca, Spain; 2 Instituto de Investigación Biomédica de Salamanca (IBSAL), Salamanca, Spain

**Keywords:** gingivitis, meta-analysis, olive oil, periodontal treatment, periodontitis, randomized clinicaltrials, systematic review

## Abstract

**Background/objectives:**

Periodontal diseases, which are highly inflammatory in nature, are very common throughout the world. In recent years, natural products have gained special attention as a complement to conventional therapy, and olive oil/ozonated olive oil (OLO/OzOLO), due to its anti-inflammatory and antimicrobial properties, has been proposed for these treatments. The aim of our study was to demonstrate its short-term clinical efficacy in periodontal treatment.

**Methods:**

This systematic review and meta-analysis was conducted in accordance with Cochrane guidelines, and searches were performed in PubMed, Embase, Cochrane Central, Scopus, and Web of Science (WOS) to identify eligible studies. Review Manager 5.4.1 and SPSS Statistics 30.0® were used to calculate standardized mean differences (SMD) and 95% confidence intervals (CI). The main outcomes assessed for periodontitis were probing depth (PPD), bleeding on probing (BoP), and clinical attachment level (CAL), and for gingivitis, plaque index (PI), gingival index (GI) and bleeding index (BI).

**Results:**

Twelve randomized clinical trials (RCTs) involving 456 subjects were included. In periodontitis, compared with controls, OLO/OzOLO improved BoP reduction in the medium term (8–12 weeks) [−0.66; 95% CI (−1.07 to −0.26); *p* = 0.001], and no benefits were observed in terms of PPD reduction and CAL gain. In gingivitis, the effect of OLO/OzOLO, compared to controls, produced a significant reduction in all three indices (PI, GI, BI) after 2–8 weeks [−1.52, 95% CI (−2.60 to −0.44); *p* = 0.006].

**Conclusions:**

Despite limitations, OLO/OzOLO treatments result in short-term CAL gain and improved gingival parameters.

**Systematic Review Registration:**

doi: 10.37766/inplasy2025.10.0065, Identifier INPLASY 2025100065.

## 1 Introduction

In 2021, global periodontal disease cases reached 951.3 million (95% Uncertainty Interval, UI: 729.0–1,183.3 million). The World Health Organization (WHO) has highlighted that oral diseases, including periodontal conditions, affect nearly 3.5 billion people worldwide, with periodontal disease being one of the most prevalent chronic inflammatory conditions. If left untreated, they destroy the dental support apparatus, leading to tooth loss and economic and social repercussions for those who suffer from them (1, 2).

Periodontal disease begins as gingivitis, the most common and reversible form of the disease. It is characterized by inflammation of the marginal and attached gingiva as an inflammatory response to an imbalance, or dysbiosis, in the subgingival biofilm. The progression from gingivitis to the destructive form of the disease, periodontitis, is a multifactorial process that does not occur universally. It requires the persistence of a dysbiotic biofilm together with a dysregulated immune response by the host or the existence of certain systemic and environmental risk factors (such as smoking or diabetes) (3–6).

Although mechanical debridement by scaling and root planing (SRP) has been considered the gold standard for non-surgical periodontal treatment for 65 years, certain factors, such as the anatomical position of the teeth, deep periodontal pockets, and the invasive capacity of some bacteria in the tissues, reduce its effectiveness (7). In these cases, invasive techniques such as flap surgery are necessary to expose the affected areas. However, a number of difficulties lead to incomplete removal of bacterial reservoirs, allowing microorganisms to survive and microbial activity to continue in these (8). These drawbacks and the rigorous clinical protocols required for these treatments can lead to limitations in clinical practice, as well as negative results such as gingival recession, enamel loss, and tooth sensitivity (9). All of this has led to the use of various antimicrobial agents, either systemically or locally, as a complement to SRP (10, 11). Local application has more advantages than systemic use, mainly because the product remains in the affected area for longer, systemic absorption is reduced, and, above all, the development of resistant bacterial strains is significantly reduced (12, 13).

In recent years, natural adjuvant products have shown good results in surgical and non-surgical periodontal treatment (14), and there are an increasing number of studies focusing on phytopharmaceuticals to obtain antimicrobial, antiseptic, anti-inflammatory, and antioxidant effects in periodontal diseases (15, 16).

Polyphenols are natural, synthetic, or semi-synthetic organic micronutrients with multiple phenolic groups in their structure, possibly making them the largest group of chemicals in the plant kingdom (17). Olive olil (OLO) is rich in these molecules (especially biophenols and tocopherols), with powerful anti-inflammatory and antioxidant properties. These micronutrients have beneficial effects on inflammatory markers and endothelial function, showing promise in the treatment of autoimmune and inflammatory diseases (18).

**Figure 1:**
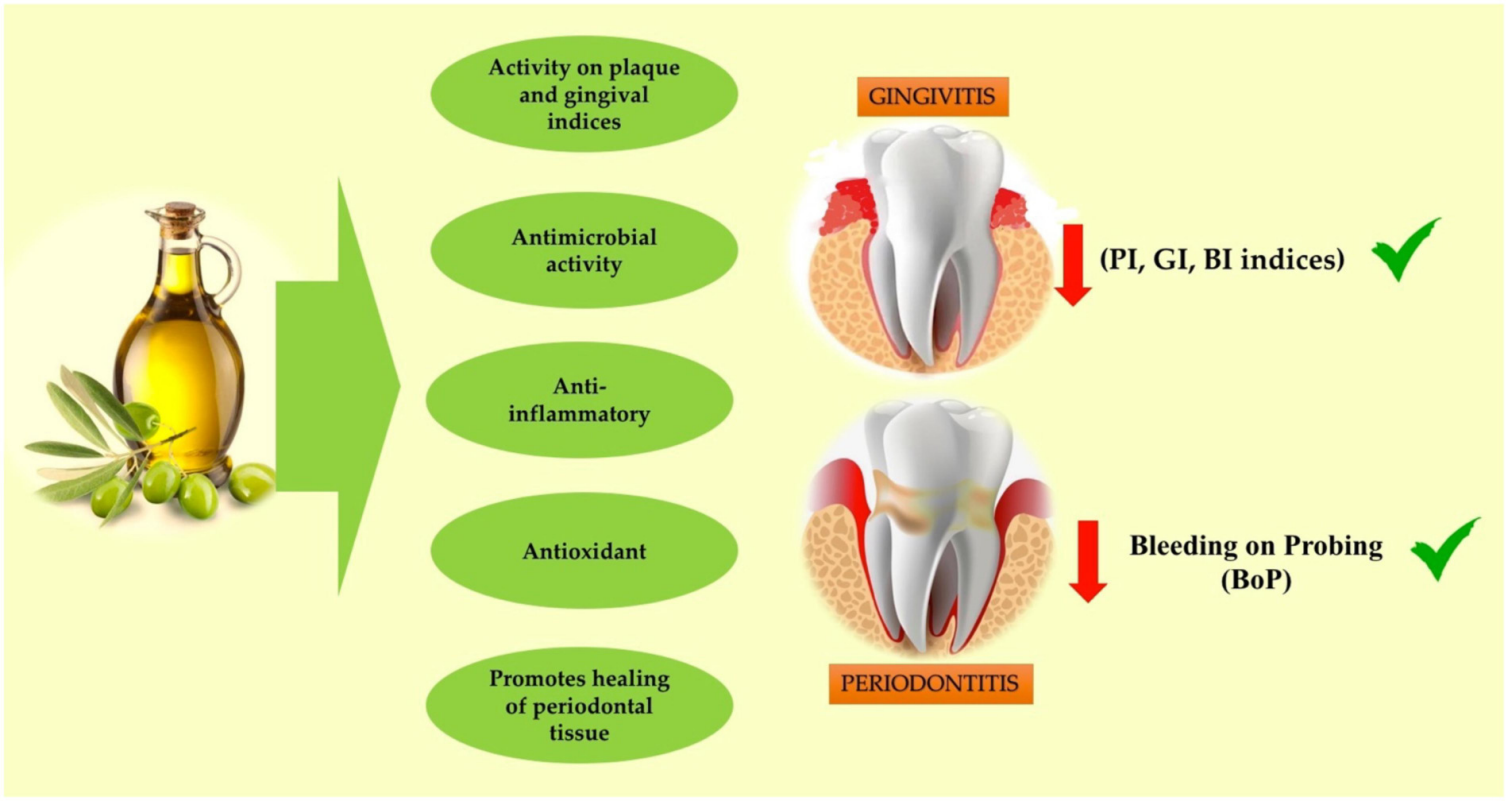
Olive oil and its properties against periodontal diseases. (PI, plaque index; GI, gingival index; BI, bleeding index).

OLO is obtained from the fruit of the olive tree and, according to European Union regulations, is classified into four types: extra virgin OLO; virgin OLO; OLO; and OLO pomace (19). It has antimicrobial activity against a wide spectrum of microorganisms, including Gram-positive and Gram-negative anaerobes (19). Massage with olive oil gum reduces the count of *Streptococcus mutans* and *Lactobacillus*, as well as plaque and gingival indices, so it can be used as a preventive agent to maintain and improve oral health, due to components other than fatty acids (20, 21). On the other hand, the abundance of monounsaturated fatty acids and phenolic compounds in OLO provides, in addition to anti-inflammatory benefits, antioxidant benefits that promote periodontal tissue healing, reducing oxidative stress (OS) in its environment (Figure 1).

Furthermore, its natural origin and biocompatibility minimize the risk of adverse reactions in tissues, making it suitable for repeated applications (22). Previous preclinical studies have shown that in animal models with aged periodontium (24-month-old rats), where periodontal diseases are more common, age-related alveolar bone loss (a characteristic of periodontal disease) can be treated with an appropriate diet (23). Specifically, animals fed olive oil showed better modulation of inflammation, homeostasis, and OS (24).

OzOLO is a product resulting from the application of ozone and has specific capabilities due to the combination of the antimicrobial effects of ozone and the anti-inflammatory and emollient properties of olive oil, making it a treatment option for periodontal diseases, where it can offer sustained antimicrobial action, promote tissue healing, and reduce dependence on other antimicrobials (25).

For all these reasons and given the qualities of OLO/OzOLO and the scarcity of meta-analyses on the subject, our study aimed to draw solid conclusions about the clinical efficacy of OLO (any type)/OzOLO (any type) in the treatment of periodontal diseases, based on randomized clinical trials (RCTs), with the aim of providing clinicians with useful evidence-based decision-making information regarding complementary treatment for periodontal diseases and, at the same time, guiding future research efforts.

## 2 Methods

### 2.1 Protocol and registration

The protocol for this review was conducted and structured in accordance with the PRISMA (Preferred Reporting Items for Systematic Reviews and Meta-Analysis) statement (26) and the Cochrane Handbook guidelines (27). The review was registered in INPLASY with the registration number INPLASY2025100065 (doi: 10.37766/inplasy2025.10.0065).

### 2.2 PICOs and focused question

The specific question used for the literature search was structured according to the PICOS format (participants, interventions, control, outcomes, study design): “Is OLO/OzOLO treatment effective as a single or complementary therapy for the treatment of gingivitis/periodontitis in adult patients?”

Intervention studies in adult patients with periodontal disease (gingivitis or periodontitis) (P) were included, comparing treatment with OLO/OzOLO, as a complementary or basic treatment (I), with conventional treatment, placebo, or no treatment (C) to observe the effects on periodontal clinical parameters (O), and only randomized clinical studies were considered (Table 1).

**Table 1:** PICOs format.

Population	Adult subjects with gingivitis or periodontitis
Intervention	OLO (any type)/OzOLO (any type) as a single treatment or adjuvant.
Comparisons	Conventional treatment, placebo, or no treatment
Outcomes	Observe the effects on periodontal clinical parameters (*Δ* PI; *Δ* BI; *Δ* GI; *Δ* PPD; *Δ* CAL; *Δ* BoP)
Study design	RCTs with at least 10 patients

PI, plaque index; GI, gingival index; BI, bleeding index; PPD, pocket probing depth; CAL, clinical attachment level; *Δ*, variable alteration; RCTs, randomized clinical trials.

### 2.3 Studies selection; inclusion and exclusion criteria

The original research studies were selected according to the following inclusion criteria: (i) RCTs (single or double blind) with more than 10 participants (*n*≥) aged 18 years or over; (ii) that treated periodontal diseases; (iii) that provided data on clinical parameters sufficiently indicative of periodontal disease; (iv) that used statistical methods, including means and standard deviations (SD), together with units of measurement of mediator levels; (v) without language restriction. Studies that did not meet all the criteria, lacked data on periodontal disease, cases of advanced periodontitis requiring periodontal surgery, were experimental studies in animals or *in vitro*, clinical cases or case series with fewer than 10 patients, literature reviews, and irrelevant studies (editorials, conference contributions, historical reviews, etc.) were excluded.

### 2.4 Search approach

Two reviewers (NL-V, AL-V) independently searched PubMed through the Medline, Embase, Scopus, and Cochrane Central databases, as well as the Web of Science (WOS) scientific information service, up to June 30, 2025, using Medical Subject Headings (MeSH) terms and keywords: “olive oil” [MeSH terms] OR “olive oil/therapeutic use” [All fields] AND “polyphenols/therapeutic use” [All fields] AND “humans” [MeSH terms] for Scopus; “olive oil” [MeSH terms] OR “olive oil/therapeutic use” [All fields] AND “polyphenols/therapeutic use” [All fields] AND “Antioxidants/therapeutic use” AND “gingivitis” [MeSH terms] OR “periodontitis” [All fields] OR “periodontal diseases” [MeSH terms] for PubMed-Medline; “gingivitis” OR “periodontitis” [Title/Abstract] AND “olive oil” [Title/Abstract]; “gingivitis” [MeSH terms] OR “periodontitis” OR “periodontal diseases” [MeSH terms] AND “olive oil” [MeSH terms] for Embase; “olive oil” AND “gingivitis” OR “periodontitis” OR “periodontal diseases” AND “humans” for Cochrane Central; and “olive il/pharmacology” OR “olive oil” AND “gingivitis” OR “periodontitis” OR “periodontal diseases” AND “humans” for WOS.

In addition, a manual search was performed, and grey literature was consulted (Teseo, SciELO, ProQuest, and Google Scholar, focusing on the first 250 results of this search engine); the bibliographic references of the selected studies were also consulted to obtain as much information as possible. No restrictions were applied in terms of language, geographical location, or time periods (Table 2).

**Table 2:** Strategy and search chains.

Databases	Search details
PubMed via Medline	“olive oil” [MeSH terms] OR “olive oil/therapeutic use” [All fields] AND “polyphenols/therapeutic use” [All fields] AND “Antioxidants/therapeutic use” AND “gingivitis” [MeSH terms] OR “periodontitis” [All fields] OR “periodontal diseases” [MeSH terms]
Embase	“gingivitis” OR “periodontitis” [Title/Abstract] AND “olive oil” [Title/Abstract]; “gingivitis” [MeSH terms] OR “periodontitis” OR “periodontal diseases” [MeSH terms] AND “olive oil” [MeSH terms]
Cochrane Central	“olive oil” AND “gingivitis” OR “periodontitis” OR “periodontal diseases” AND “humans”
Web of Science	“olive il/pharmacology” OR “olive oil” AND “gingivitis” OR “periodontitis” OR “periodontal diseases” AND “humans”
Scopus	“olive oil” [MeSH terms] OR “olive oil/therapeutic use” [All fields] AND “polyphenols/therapeutic use” [All fields] AND “humans” [MeSH terms]
Boolean operators	AND and OR

### 2.5 Data extraction

Two reviewers (NL-V and AL-V) extracted and tabulated the data from each included study using the standardized data extraction tool “The Joanna Briggs Institute Me-ta-Analysis of Statistics Assessment and Review Instrument” (JBI-MAStARI) (28). They then reviewed the titles and abstracts of the preselected studies. Those that met the inclusion criteria were read and analyzed in full, and the data they provided were extracted. Discrepancies between reviewers were resolved through discussion. Cohen's kappa (*κ*) index (29) was used to assess agreement between assessors. Data extracted from the studies included specific details of the populations, methods, specific objectives, and results relevant to the question of interest. They were double tabulated (one tabulation per reviewer) to minimize error bias.

### 2.6 Data analysis

The main outcomes of the intervention used in the meta-analysis were changes in probing depth (PPD), bleeding on probing (BoP), and clinical attachment level (CAL) from baseline, one, three and six months after treating periodontal pockets with non-surgical therapy. All analyses were based on the mean difference (MD) and SD to estimate continuous data, and on 95% confidence intervals (CI) to evaluate categorical data between treatment and controls, using the random effects model for continuous outcomes of BoP, PPD reduction, and CAL gain (30). The results were presented in the form of forest plots, and heterogeneity between studies was assessed using the *I*^2^ statistic (*I*^2^ ≥ 50% indicates substantial heterogeneity). The threshold for statistical significance was set at *p* < 0.05. Due to the heterogeneity of the results, a random effects meta-analysis was performed. All statistical analyses were performed using Review Manager software (RevMan Software. Version 5.4.1; The Cochrane Collaboration, Copenhagen, Denmark; 2020).

The alternative research hypothesis of this study was that there are differences in treatment outcomes between the intervention group (with OLO/OzOLO) and the control group.

### 2.7 Risk of bias

The risk of bias was assessed by two evaluators (NL-V and AL-V) independently, using the Cochrane Risk of Bias Tool (RoB2, version of August 22, 2019) (31), which uses seven domains: random sequence generation (selection bias); allocation concealment (selection bias); blinding of participants and personnel (performance bias); blinding of outcome assessment (detection bias); incomplete outcome data (attrition bias); selective reporting (reporting bias); other biases. The certainty of the evidence was assessed using the GRADE approach. Since all studies were RCTs, the initial rating was set as “high” and then adjusted downward considering factors that reduce certainty, such as a high risk of bias. However, the downgrading of certainty can be offset by factors that can increase it, such as a large effect size. Discrepancies between assessors were discussed in order to reach a consensus.

## 3 Results

### 3.1 Search results

**Figure 2:**
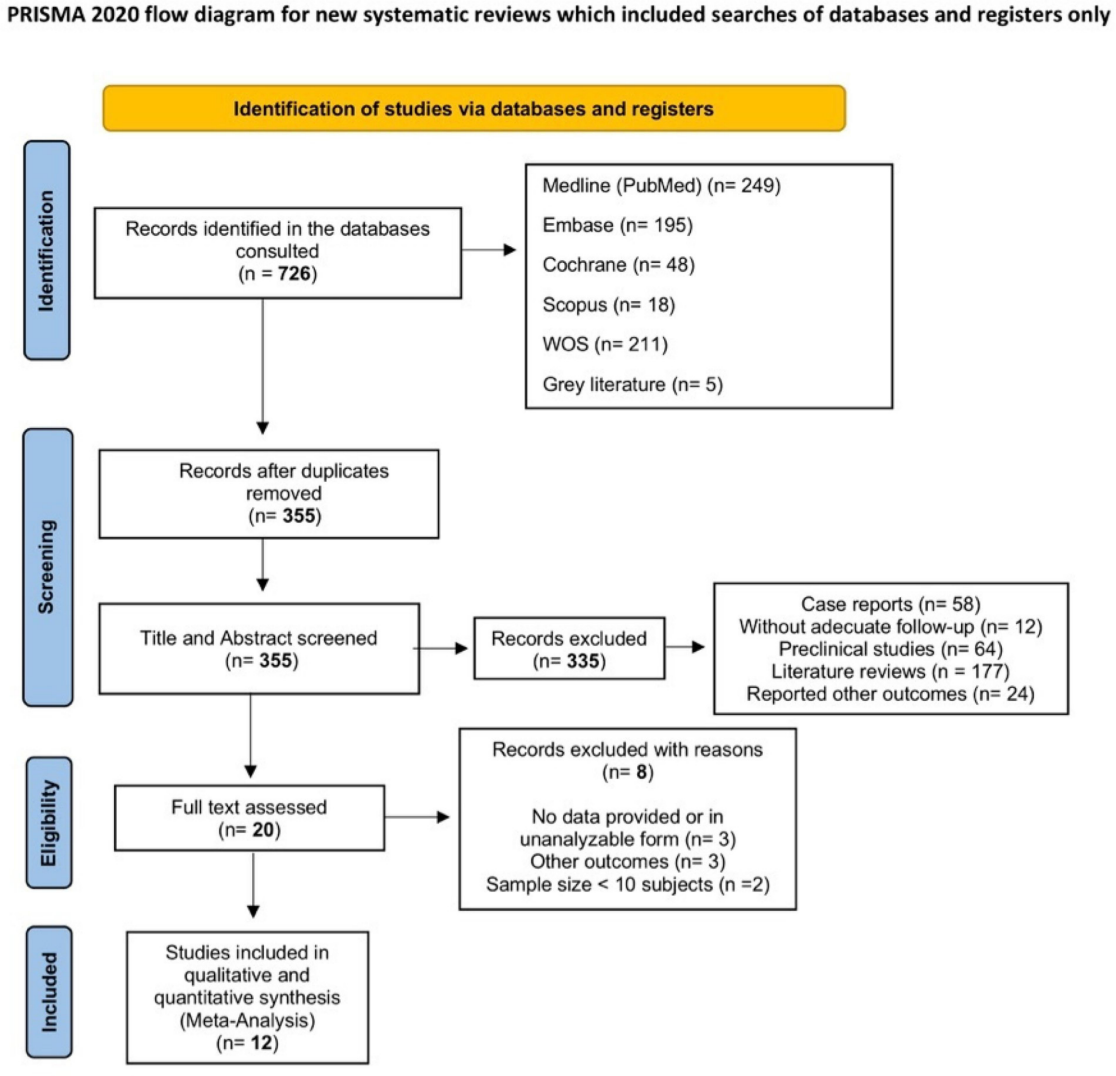
Flow diagram.

A total of 726 studies were identified in the databases. After removing duplicates, 355 studies were eligible for evaluation. A total of 336 studies were excluded during the title and abstract screening process. These studies were case series, did not have adequate follow-up, were preclinical studies, were literature reviews, or reported other outcomes. Twenty full publications were assessed for eligibility. After completing the eligibility assessment, eight studies were excluded (Table 3), and 12 studies were included in this review (32–43) (search flow diagram: Figure 2).

**Table 3:** Excluded studies and reason for exclusion.

Study	Reasons for exclusion
López-López et al. (2025)	No periodontal parameter data was provided.
Singh et al. (2024)	The study focused on the effect of ozone vs. chlorhexidine on chronic periodontitis.
Lendhey et al. (2020)	The data was provided in the form of graphs.
Karim et al. (2022)	Other outcomes
Musa et al. (2022)	Other outcomes
Grassi et al. (2025)	Did not provide analyzable data.
Pruthi et al. (2024)	The sample size was less than 10 subjects.
Kaur et al. (2024)	The sample size was less than 10 subjects.

### 3.2 General and specific characteristics of the included studies

Seven clinical trials with a sample of 236 subjects (32, 34–38, 41) evaluated the efficacy of OLO/OzOLO in the treatment of periodontitis, and five, with a sample of 220 subjects, evaluated its efficacy in the treatment of gingivitis (33, 37, 39, 40, 42). The largest samples were provided by the studies by Mukherjee et al. (39) on periodontitis and Zumbo et al. (38) on gingivitis. The longest follow-up was in the study by Nardi et al. (35) on periodontitis and Rodríguez-Agurto et al. (40) on gingivitis. Nine of the 12 included studies were conducted in India (75%) (32–34, 36, 37, 40–43), and none of them reported adverse effects. Only two studies received funding (33, 40) (Tables 4, 5).

**Table 4:** General characteristics of the included studies.

Author, year	Study design	Participants control/experimental	Clinical parameters control/experimental	Intervention	Follow-up	Outcomes
Patel et al. (32)	Split mouth	20 subjets (10/10)	(4 weeks) Average PPD mm Control 5.04 ± 0.74 Experimental 6.01 ± 0.373 Average CAL mm Control 5.26 ± 0.74 Experimental 6.04 ± 0.36 Average BoP (%) Control 2.77 ± 0.16 Experimental 2.24 ± 0.29 ______________ (8 weeks) Average PPD mm Control 3.82 ± 0.376 Experimental 4.09 ± 0.38 Average CAL mm Control 3.87 ± 0.35 Experimental 4.21 ± 0.55	OzOLO	2, 4, 6, 8 weeks	PI, GI, PPD, CAL, BoP
Indurkar et al. (33)	Parallel groups	20 subjets (10/10)	Average PI Control 2.313 ± 0.47 Experimental 2.027 ± 0.32 Average GI Control 2.138 ± 0.34 Experimental 1.791 ± 0.39	OzOLO	3 weeks	PI, GI
Khare et al. (34)	Split mouth	20 subjets (10/10)	(3 weks) Average PPD (mm) Control 4.60 ± 0.699 Experimental 3.20 ± 0.632	OzOLO	1, 2, 3 weks	PI, GI, PPD
Nardi et al. (35)	Case-control	96 subjects (48/48)	(4 weeks) Average PPD (mm) Control 2.206 ± 0.781 Experimental 2.15 ± 0.922 Average BoP (%) Control 14.25 ± 7.930 Experimental 12.67 ± 5.926 _______________ (24 weks) Average PPD (mm) Control 2.451 ± 0.746 Experimental 2.083 ± 0.814 Average BoP (%) Control 8.250 ± 4.190 Experimental 7.00 ± 3.377	OzOLO	2, 4, 24weks	PI, PPD, BoP
Nambiar et al. (36)	Split mouth	30 subjects	Average PPD (mm) Control 4.56 ± 0.57 Experimental 4.19 ± 0.78 Average CAL (mm) Control 6.89 ± 0.75 Experimental 6.33 ± 0.92 Average BoP (%) Control 0.00 ± 0.000 Experimental 0.00 ± 0.00	OzOLO	12 weks	PPD, CAL, BoP
Vijeyakumar et al. (37)	Split mouth	30 subjects	Average PPD (mm) Control 3.03 ± 0.18 Experimental 2.83 ± 0.59 Average CAL (mm) Control 2.14 ± 0.12 Experimental 1.43 ± 0.29	OzOLO	3 weks	PPD, CAL
Zumbo et al. (38)	Parallel groups	75 subjects (25/25/25)	Average PI Control 0.96 ± 0.200 Experimental 0.56 ± 0.507 Average BI Control 0.84 ± 0.374 Experimental 0.44 ± 0.501	OLO	30 days	PI, BI
Mukherjee et al. (39)	Parallel groups	120 subjects (40/40/40)	(4 weks) Average PPD (mm) Control 5.28 ± 0.45 Experimental 5.40 ± 0.50 Average CAL (mm) Control 5.28 ± 0.45 Experimental 5.00 ± 0.00	OzOLO	1, 2, 4 weeks	GI, PPD, CAL
Rodríguez-Agurto et al. (40)	Parallel groups	61 subjets (20/20//21)	Average PI Control 24 ± 13 Experimental 26 ± 14 Average BI Control 20 ± 9 Experimental 22 ± 11	OLO	2, 4 months	PI, BI
Swarna Meenakshi and Subasree (41)	Parallel groups	24 subjets (12/12)	Average PI Control 1.83 ± 0.29 Experimental 0.57 ± 0.16 Average GI Control 1.88 ± 0.23 Experimental 0.96 ± 0.21	OLO	1 month	PI, GI
Patel et al. (42)	Parallel groups	20 subjets (10/10)	Average PPD (mm) Control 5.30 ± 0.48 Experimental 5.60 ± 0.69 Average CAL (mm) Control 7.60 ± 0.51 Experimental 7.20 ± 0.63 Average BoP (%) Control 1.60 ± 0.51 Experimental 1.70 ± 0.48	OzOLO	12 weks	PI, PPD, CAL, BoP
Yadav and Agarwal (43)	Parallel groups	60 subjets (30/30)	Average PI Control 1.98 ± 0.27 Experimental 2.02 ± 0.25 Average PI Control 0.65 ± 0.17 Experimental 0.74 ± 0.18	OzOLO	2 weks	PI, GI

PI, plaque index; GI, gingival index; BI, bleeding index; PPD, pocket probing depth; CAL, clinical attachment level; BoP, bleeding on probing; OLO, olive oil; OzOLO, ozonated olive oil.

**Table 5:** Specific and sociodemographic characteristics.

Study	Country	Pathology	Diagnostic criteria	Journal	Gender	Tobacco smokers	Dropouts	Adverse events	Site and funding	Financial support and sponsorship
Patel et al. (32)	India	Chronic periodontitis	PPD >6 mm; PI, Quigley Hein Index; GI, Löe and Silness Index; BoP, Muhlemann and Son Index; PPD and CAL, periodontal probe	Minerva Stomatol.	8 females	Non-smokers	No dropouts	NR	Research Center	NR
Indurkar et al. (33)	India	Gingivitis	PI, Turesky-Gilmore-Glickman modification of Quigely Hein Index; GI, Löe and Silness Index	J Indian Soc Periodontol	Group 1: 6 females Group 2: 4 females	NR	No dropouts	NR	University Center	Financial support: Ozone Forum of India and ICPA Health Product Ltd.
Khare et al. (34)	India	Chronic periodontitis	PPD ≥4 mm	J Dent Specialities	NR	Non-smokers	No dropouts	NR	University Center	None
Nardi et al. (35)	Italy	Periodontitis	PI ≥ 35%; GI ≥35%	Int. J. Environ. Res. Public Health	NR	Non-smokers	No dropouts	NR	University Center	None
Nambiar et al. (36)	India	Chronic periodontitis	PPD ≥ 5 mm; CAL ≥3 mm	J Pharm Bioall Sci	NR	Non-smokers	No dropouts	NR	University Center	None
Vijeyakumar et al. (37)	India	Chronic periodontitis	PI and GI, Silness and Löe Index; PPD and CAL Ramfjord Index	International Journal of Research and Review	NR	NR	No dropouts	NR	University Center	None
Zumbo et al. (38)	Italy	Gingivitis	GI, Silness- Löe Index; PI, O’Leary Index; BoP, Ainamo and Bay Index	J Clin Med	Group A: 14 females Group B: 19 females Group C: 11 females	Group A: 8 Group B: 6 Group C: 9	No dropouts	NR	University Center	None
Mukherjee et al. (39)	India	Chronic periodontitis	PPD ≥5 mm	J Pharm Bioall Sci	20 females	Non-smokers	No dropouts	NR	University Center	None
Rodríguez- Agurto (40)	Spain	Gingivitis	PI, Tonetti Index; GI, Ainamo and Bay Index	Scientific Reports	Goup 1: 61.9% females Goup 2: 80% females	Non-smokers	No dropouts	NR	University Center	Financial support: University of Granada (Spain) and Mucosal Innovations S.L. (Madrid,Spain).
Swarna Meenakshi and Subasree (41)	India	Gingivitis	GI, William's probe	Cureus	NR	Non-smokers	No dropouts	NR	University Center	None
Patel et al. (42)	India	Periodontitis	PPD ≥ 5 mm; CAL ≥ 3 mm; GI, Silness- Löe Index	Cureus	NR	Non-smokers	No dropouts	They mention possible adverse effects but do not indicate them.	University Center	None
Yadav and Agarwal (43)	India	Gingivitis	NR	J. Life Sci.Biotechnol. Pharma.Res	NR	NR	No dropouts	They mention possible adverse effects but do not indicate them.	University Center	NR

PPD, pocket probing depth; PI, plaque index; GI, gingival index; CAL, clinical attachment level; BoP, bleeding on probing; NR, does not report.

### 3.3 Results of the meta-analysis for non-surgical therapy and OzOLO in periodontitis

#### 3.3.1 PPD reduction

**Figure 3:**
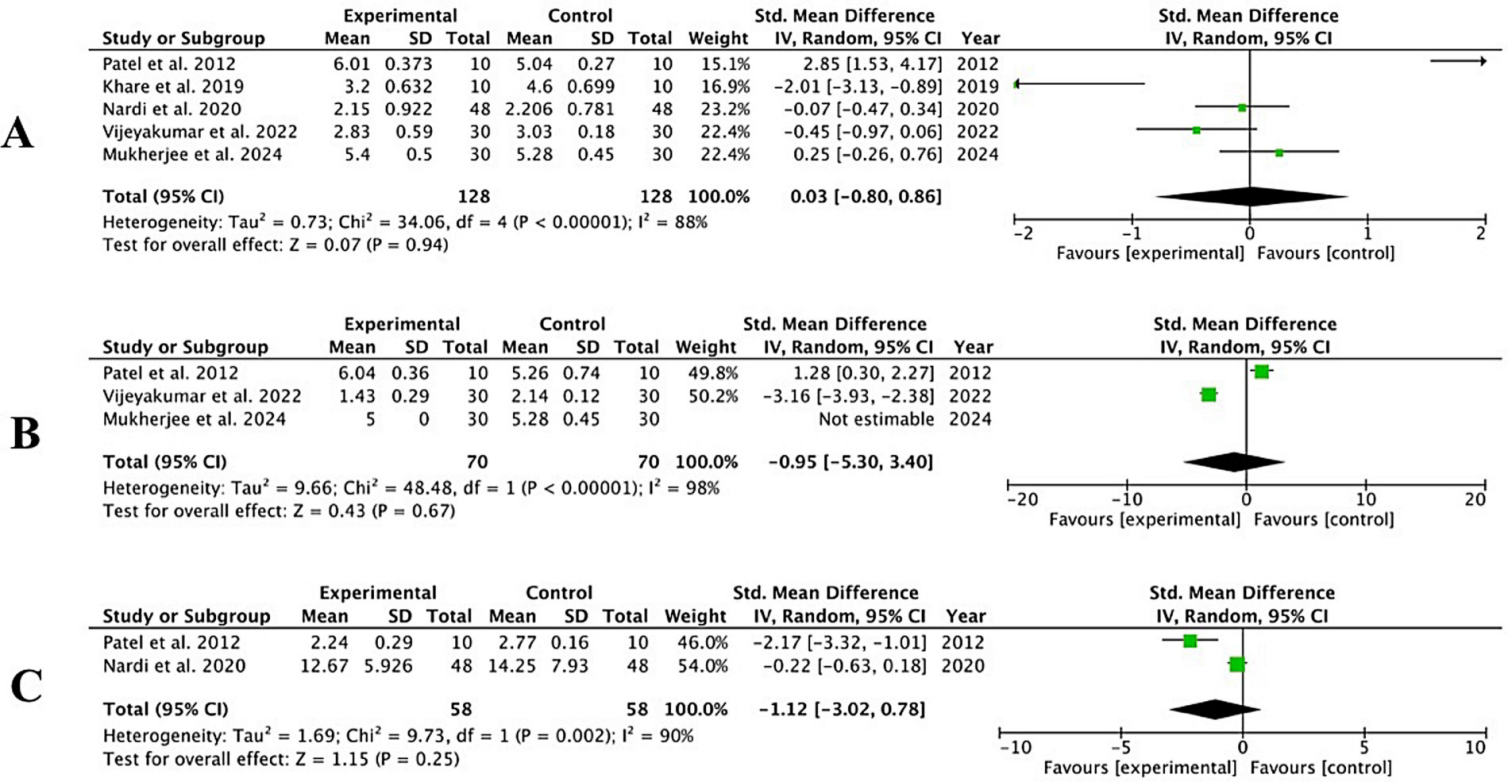
Forest plot for PPD (probing dept) reduction at 3–4 weeks **(A)** (32, 34, 35, 37, 39); 8–12 weeks **(B)** (32, 37, 39) and 12–24 weeks **(C)** (32, 35).

Five studies (32, 34, 35, 37, 39) reported a reduction in PPD at sites treated with OzOLO compared to a control group in the short term (3–4 weeks), three in medium term (8–12 weks) (32, 37, 39) and two (32, 35) in the long term (12–24 weeks). During the periods analyzed, heterogeneity was high (*I*^2^ = 88%–98%) and the meta-analysis showed no reduction in PPD in these time periods in the experimental groups compared to the controls: −0.03; 95% CI [−0.80 to 0.86]; *p* = 0.94 (3–4 weeks); −0.95; 95% CI [−5.30 to 3.40]; *p* = 0.67 (8–12 weeks) and −1.12; 95% CI [−3.02 to 0.78]; *p* = 0.25 (12–24 weeks) (Figures 3A–C).

#### 3.3.2 CAL gain

**Figure 4:**
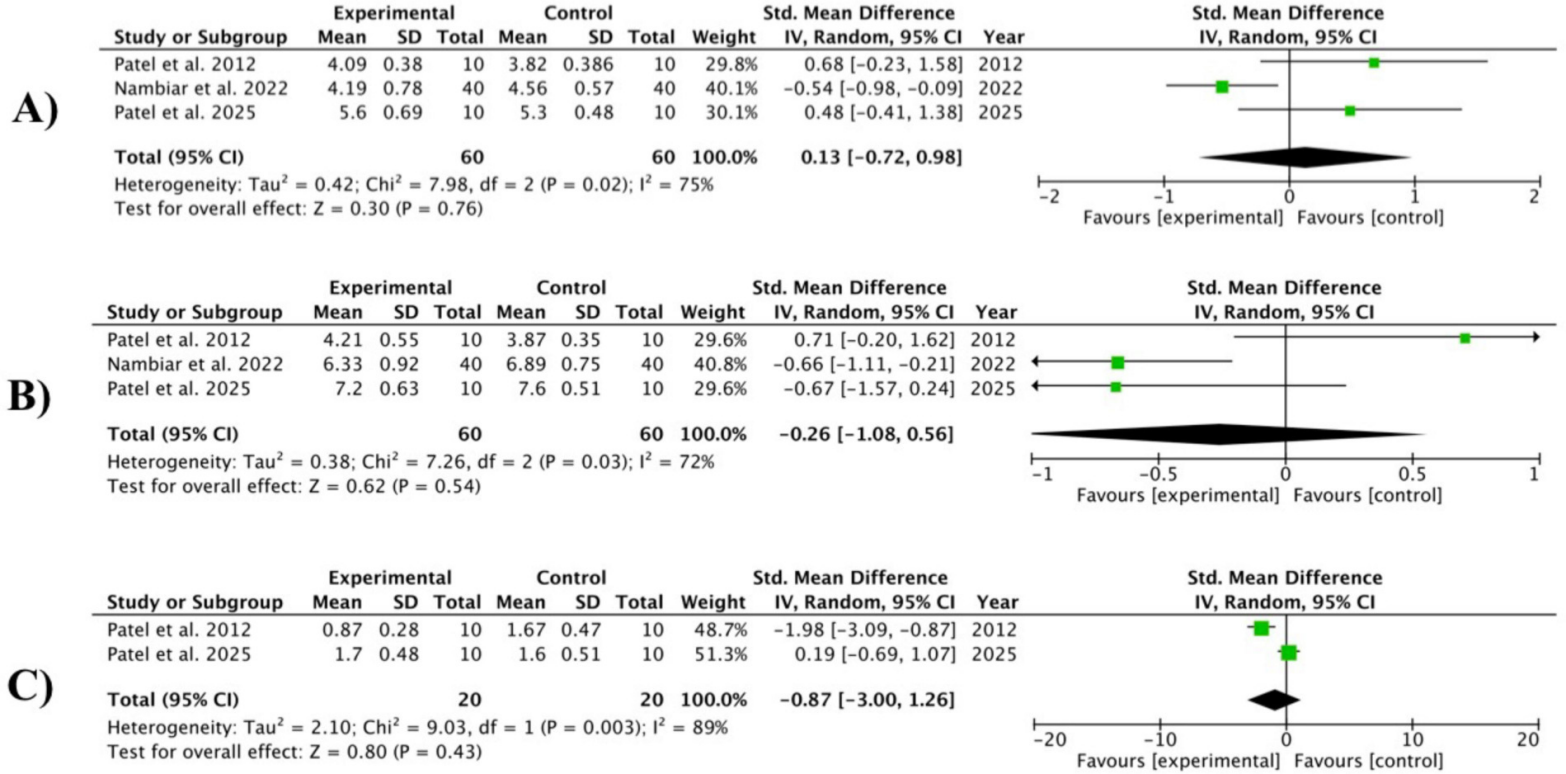
Forest plot for CAL (clinical attachment level) gain at 3–4 weeks **(A)**; 8–12 weeks **(B)** and 12–24 weeks **(C)** (32, 36, 42).

Three studies (32, 36, 42) provided data on short-term (3–4 weeks) and medium-term (8–12 weeks) CAL gain, and two (32, 42) provided long-term (12–24 weeks) data at sites treated with OzOLO, compared with a control group. Heterogeneity ranged from moderate (*I*^2^ = 72%) to high (*I*^2^ = 89%), and our meta-analysis found no statistical significance in CAL gain across the three time periods analyzed. 0.13; 95% CI [−0.72 to 0.98]; *p* = 0.76; −0.26; 95% CI [−1.08 to 0.56]; *p* = 0.54; −0.87; 95% CI [−3.00 to 1.26]; *p* = 0.43, respectively (Figures 4A–C).

#### 3.3.3 Bop reduction

**Figure 5:**
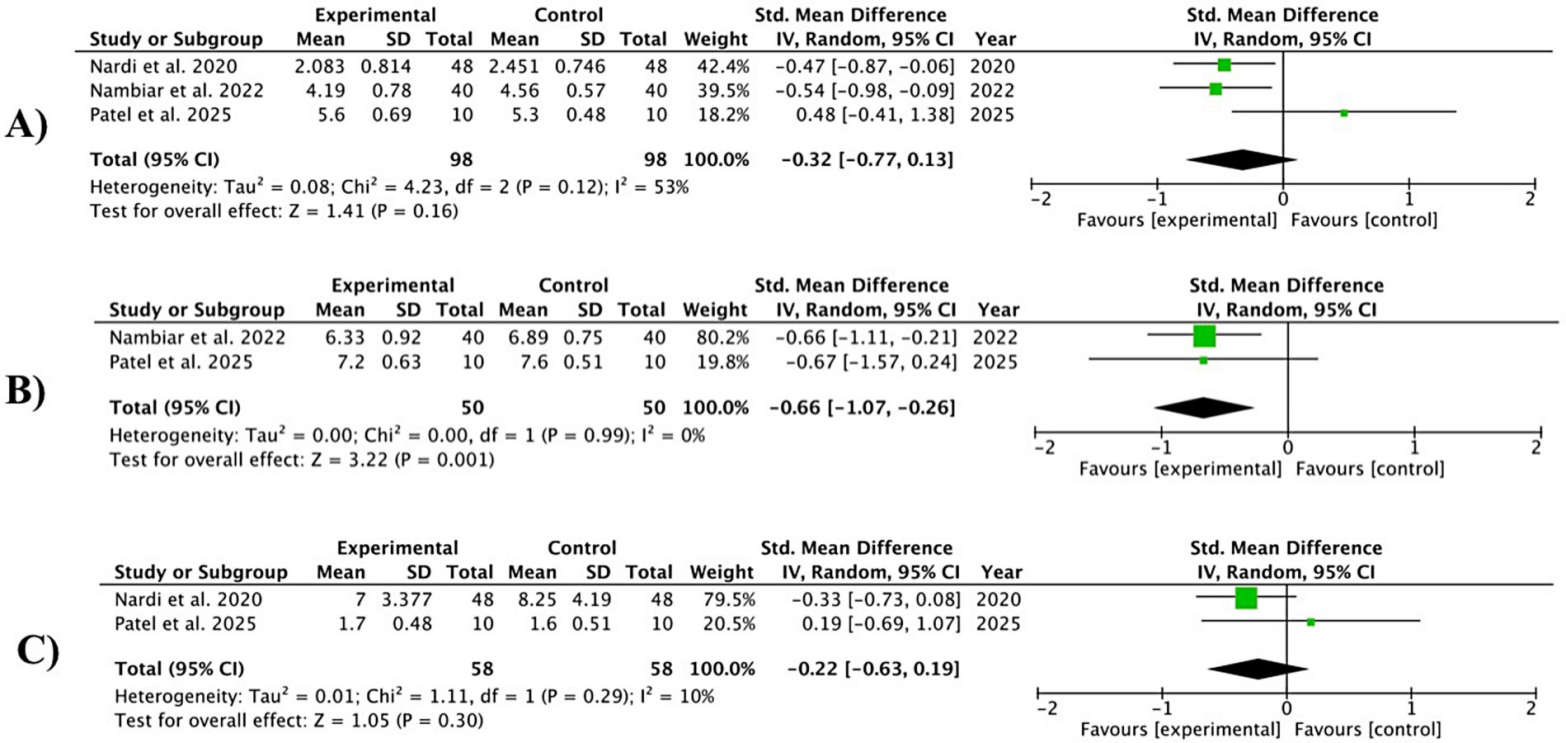
Forest plot of probe bleed reduction (BoP) at 3–4 weeks **(A)** (35, 36, 42); 8–12 weeks **(B)** (36, 42); and 12–24 weeks **(C)** (35, 42).

Three studies (35, 36, 42) presented data on BoP reduction at 3–4 weeks, two (36, 42) at 8–12 weeks, and two (35, 42) at 12–24 weeks at sites treated with OzOLO, compared with a control group. In the short term, heterogeneity was moderate (*I*^2^ = 53%) and BoP figures did not show a significant reduction [−0.32; 95% CI (−0.77 to 0.13); *p* = 0.16]. At 8–12 weeks, the heterogeneity of the studies was zero (*I*^2^ = 0%) and BoP scores were significantly reduced [−0.66; 95% CI (−1.07 to −0.26); *p* = 0.001]. In the long term (12–24 weeks), heterogeneity was low (*I*^2^ = 10%) and BoP did not show a significant reduction [−0.22; 95% CI (−0.63 to 0.19)]; *p* = 0.30 (Figures 5A, short term; 5B, medium term; 5C, long term).

### 3.4 Results of the meta-analysis for treatment with OzOLO and OLO in gingivitis

**Figure 6:**
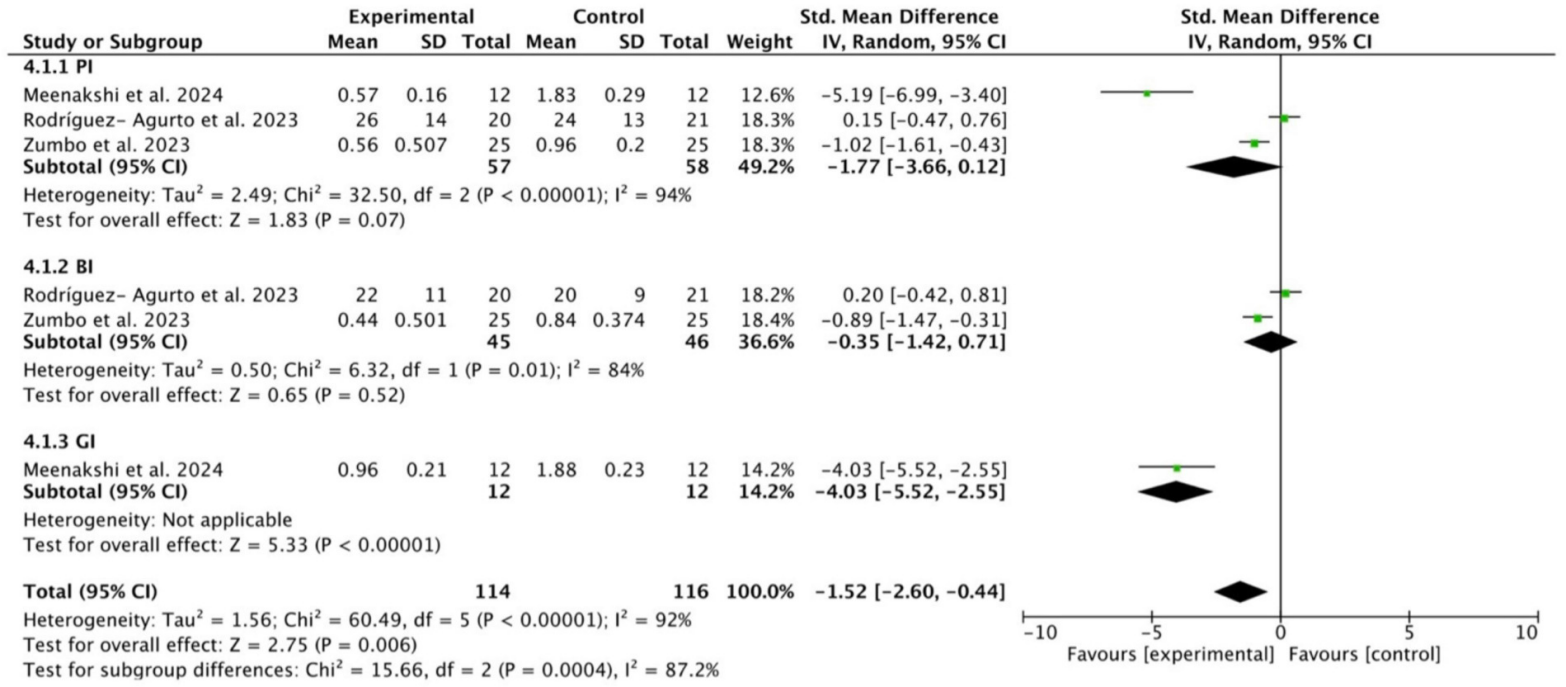
Forest plot of PI (plaque index), BI (bleeding index) and GI (gingival index) reduction at 2-8 weeks (38, 40, 41).

Three studies (38, 40, 41) provided data on the effect of OLO compared to controls on PI, BI, and GI indices over a period of 2–8 weeks. The study by Meenakshi and Subasree (41) was the only one that provided data on the effect of OLO in the treatment of gingivitis and was therefore not analyzable. We therefore resorted to a pooled analysis of studies in order to increase statistical power. Heterogeneity was high (*I*^2^ > 85%), and the overall effect showed a significant reduction was observed in all three indices [−1.52, 95% CI (−2.60 to −0.44); *p* = 0.006]. However, OzOLO was not found to be significant in reducing PI and GI (Figure 6).

### 3.5 Quality of studies. Results of the Risk of Bias Assessment

**Figure 7:**
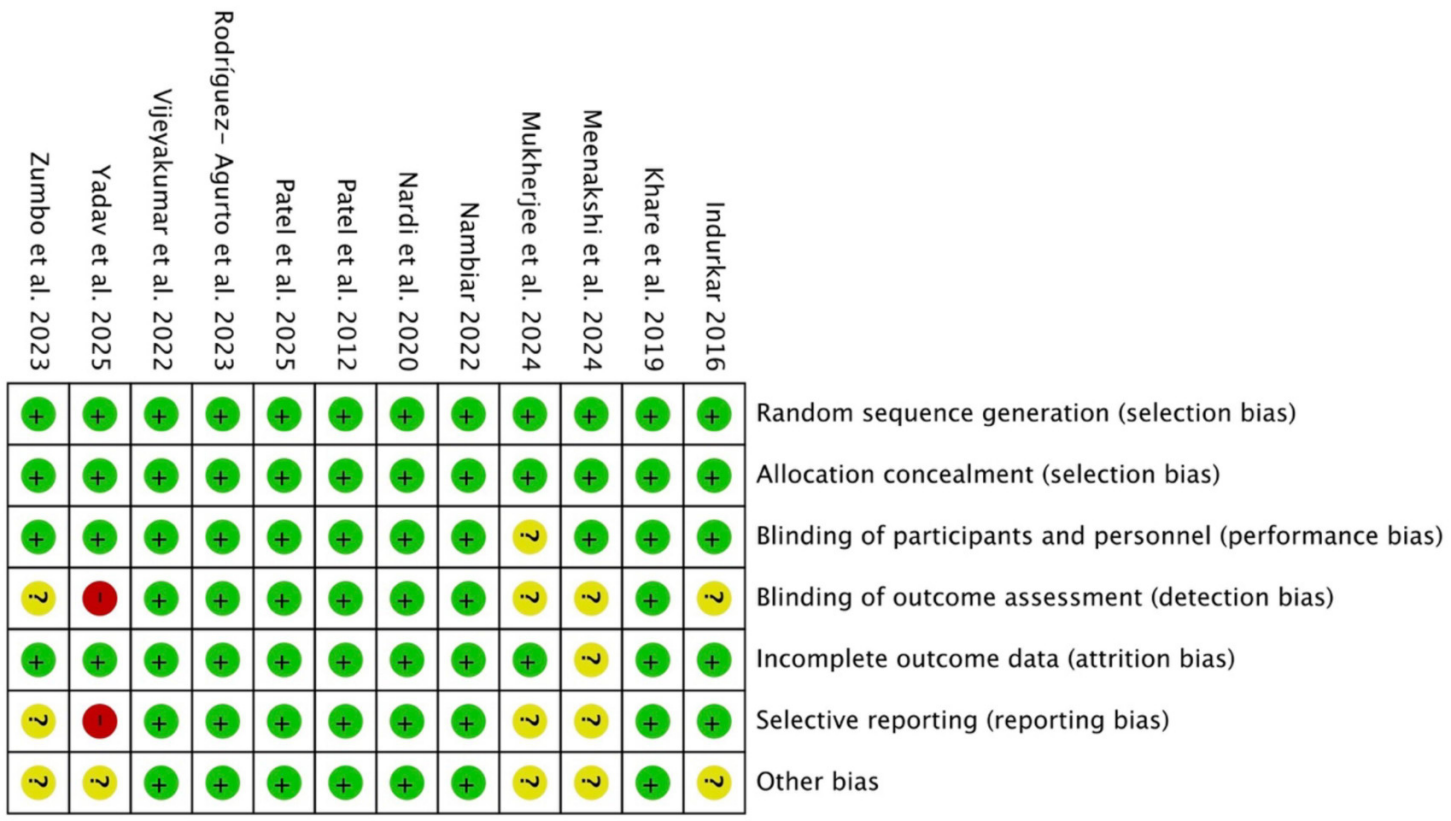
Graph of the risk of bias in the studies included in the meta-analysis, assessed using the tool (RoB2) (32–43).

**Figure 8:**
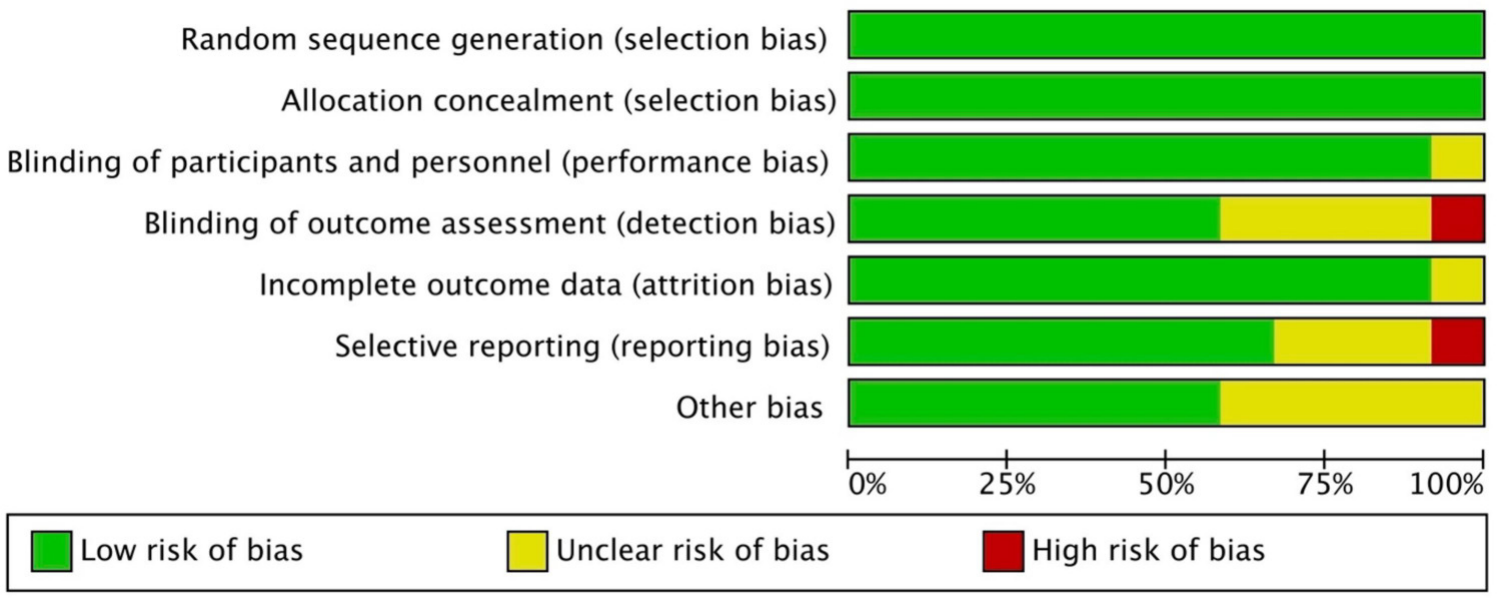
Risk of bias graph: review of authors’ judgements regarding each risk of bias item.

Risk of bias assessment is one of the pillars of evidence-based medicine, so two re-viewers (NL-V and AL-V) independently analyzed the quality of the included studies using the Cochrane Risk of Bias tool (31), and any discrepancies between them were resolved through discussion. RCTs were assessed in seven domains. Domains with a low risk of bias were rated “low,” and those considered to have a high risk of bias were rated “high.” Uncertain biases or those without information were rated “borderline.” Some studies included randomization software, and it was difficult for the reviewers to know which areas they addressed and which they did not. Overall, the included studies had a low risk of bias, and only the study by Yadav and Agarwal (43) had a high risk of bias in the domains of blinding of outcome assessment (detection bias) and selective reporting (reporting bias). These two biases, together with other bias, were the ones with the greatest lack of information. The most commonly reported biases were random sequence generation (selection bias), allocation concealment (selection bias), blinding of participants and personnel (performance bias), and incomplete outcome data (attrition bias) (Figures 7, 8).

### 3.6 Publication bias results

**Figure 9:**
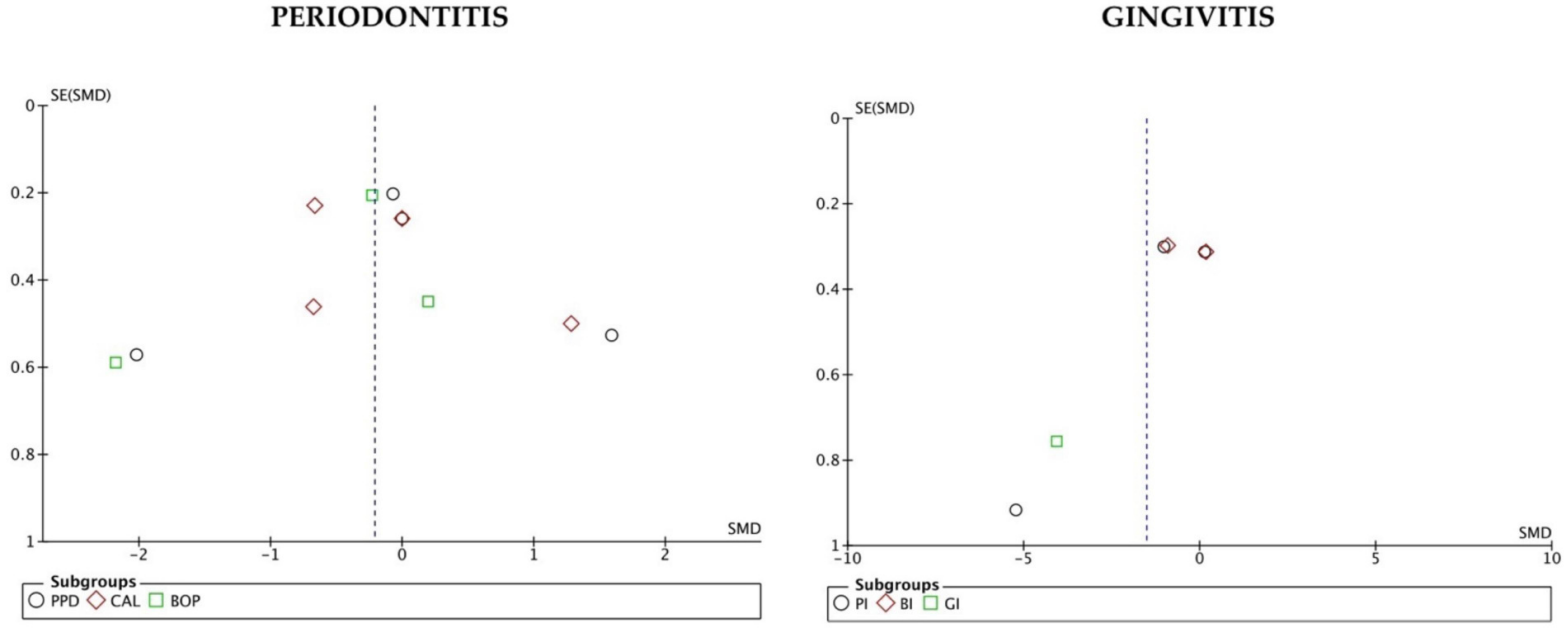
Funnel plots showed that studies on periodontitis are skewed and studies on gingivitis are symmetrical.

The funnel plot in periodontitis studies is asymmetrical, whereas in gingivitis studies the plot is more symmetrical. However, it is difficult to assess whether the asymmetry is due to publication bias, as there are several reasons that could explain it (44) (Figure 9).

## 4 Discussion

This systematic review, which includes a meta-analysis, evaluated the potential additional effects of local application of OLO/OzOLO on clinical outcomes for gingivitis and non-surgical periodontal treatment of periodontitis.

Twelve RCTs met the inclusion criteria, with follow-ups of 3–24 weeks for non-surgical treatment of periodontitis and 2–8 weeks for treatment of gingivitis.

Gingivitis is a reversible inflammatory condition caused by plaque accumulation on the tooth surface. It is characterized by redness, swelling, and bleeding of the gums, without loss of periodontal attachment and without affecting the periodontal ligament, cementum, or alveolar bone (45). The phenolic compounds present in olive leaves and fruits have been shown to have anti-inflammatory, antioxidant, and antibacterial effects (46, 47). In addition, polyphenols improve stress response by increasing antioxidants and mitochondrial protection (48). Its anti-inflammatory capacity can reduce the synthesis of pro-inflammatory cytokines TNF-α, IL-1β, IL-6, and COX-2 (49). Silva et al. (50) evaluated the effect of OLO in Wistar rats and reported that treatment with a dose of 5 mg/kg significantly decreased edema, histological damage, COX-2 expression, and inducible nitric oxide synthase, and markedly reduced the degree of bone resorption and soft tissue inflammation. In this regard, López-López et al. (22), in an RCT with 22 patients, observed that the use of a toothpaste containing OLO for 4 weeks was able to reduce the proinflammatory salivary cytokines IL-1β and TNF-α to healthy levels. These results would be consistent with those revealed in this meta-analysis, in reference to the parameters indicative of gingival inflammation PI, GI, and BI, in which we observed a significant overall reduction (*p* = 0.006) after 2–8 weeks of using OLO. If we look at a prospective case-control study conducted on 20 children aged 6–12 years (51) (published after our data collection), in which OLO was used as the study group and chlorhexidine as the control group, the mean reductions in plaque scores were not significant between the groups after 1 month; however, the OLO group achieved a greater reduction in gingival scores compared to the control group. The authors of the study recommend using OLO for 4 weeks, which showed a significant effect on gingivitis indices, and highlight that this effect could be due to the anti-inflammatory and modulating effect of olive oil on the gums. Queremos destacar que este estudio fue publicado posteriormente a la fecha de finalización de recogida de datos.

The results obtained in this study are similar to those obtained in our meta-analysis on gingivitis, in which we analyzed periods of between 2 and 8 weeks. All of this would be supported by studies suggesting that adding OLO to oral antimicrobial agents, mouthwashes, and toothpastes would reduce the adhesion of bacteria to acquired film, minimizing the reformation of dental plaque (52). Others have also suggested that the lipophilic substance present in edible oils, such as OLO, can modify the structure of the biofilm and modulate bioadhesion processes, adding hydrophobic characteristics to the tooth surface, which would benefit the maintenance of good oral health (53). In our meta-analysis, only three studies (38, 40, 41) evaluated the effectiveness of OLO in the treatment of gingivitis, with the others (33, 38) resorting to OzOLO, which could lead to bias in the interpretation of results.

The mechanism of action of OzOLO is based on the gradual release of ozone upon contact with tissues, allowing prolonged microbial control while minimizing cytotoxicity. Ozone also accelerates wound healing and improves tissue regeneration (54, 55). Karygianni et al. (56) evaluated *in vitro* the antimicrobial effect of maslinic acid and oleanolic acid, extracts obtained from the leaves of Olea Europaea (olive tree), against oral bacterial species. They reported their effectiveness against anaerobic oral microorganisms, including Gram-negative species relevant to periodontal diseases, such as *Porphyromonas gingivalis* and *Prevotella intermedia*. Other natural oils such as tea tree oil or coconut oil, used in mouthwashes, can be effective in reducing dental plaque or positively altering gingival indices, compared to chemical products such as chlorhexidine or non-surgical mechanical treatments (57, 58).

Connective tissue maturation occurs after 4 weeks, meaning that results in shorter periods are not significant, except as a follow-up to the disease's stability or progression with the treatment applied. Paterno et al. (59) highlight in a recent meta-analysis that most of the total reduction in PPD in shallow pockets occurs in the first 1–2 months, with an additional, albeit smaller, reduction up to 3–4 months after treatment. They also point out that complete maturation of connective tissue fibers appears to require much longer periods of time, exceeding six months.

All studies included in our meta-analysis that evaluated periodontitis used OzOLO (32, 34–37, 39, 42).

Thomé et al. (60), in a systematic review, included studies with all combinations of ozone (gas, water, or oil) and also reported very limited additional benefits in terms of PPD reduction. However, there are clear discrepancies in the literature regarding the benefits of ozone in the complementary treatment of periodontitis. While some studies have found no reduction in clinical parameters when ozone was used as a complement to SRP (61), a recent meta-analysis highlighted its positive effects in combination with SRP in terms of PPD and GI indices in patients with periodontitis (62).

We also found no reduction in BoP values, either in the short follow-up periods (3–4 weeks) (*p* = 0.25) or in the longer periods (12–24 weeks) (*p* = 0.30). Only in the medium-term follow-up periods (8–12 weeks) was statistical significance found (*p* = 0.001). These results would be consistent with those shown by Grassi et al. (63) (excluded from our meta-analysis because the data were not analyzable), who, in an RCT, evaluated the effects of OzOLO on periodontal pockets in stage II–IV periodontitis and found significant reductions in BoP at 12 weeks (*p* < 0.05). However, other clinical studies also found no significant improvement in clinical parameters when ozone combinations associated with SRP were used (64, 65).

The increase in clinical attachment level (CAL) is important, especially because it is the only method for clinically assessing the stability or progression of periodontal disease. In addition, it can be easily monitored over time (66, 67). In our meta-analysis we found no significant values in CAL gain in the intervention groups compared to the controls, either in the short term (3–4 weeks; *p* = 0.94), medium term (8–12 weeks; *p* = 0.67), or longer follow-up periods (12–24 weeks; *p* = 0.25). However, several studies have pointed out (68–70) that part of the CAL gain is often lost over time, usually due to patients' non-compliance with support programs.

Finally, it is worth noting the high degree of heterogeneity shown in the studies included in our meta-analysis, among whose causes the following could be mentioned: the different indices used in the evaluation of the same clinical parameter; the calibration processes between examiners and the degree of agreement between them (Kappa coefficient); the different instruments used to measure gingival and periodontal indices [six studies that evaluated periodontitis used the UNC-15 probe and one (37) did not report this]. Of those that studied gingivitis, two did not report (33, 38), one used the UNC-15 (40) and one (41) used William's probe.

## 5 Study limitations

Our meta-analysis is affected by a number of limitations, which are described below: i) Relatively low number of studies available and, therefore, limited amount/type of information collected in them; ii) networks for reducing PPD and BoP, and CAL gain were scarce, due to the low number of direct comparisons and the low number of associated studies, and some of the comparisons were not possible because they were based on a single study; iii) the estimates for most comparisons were quite imprecise, which reduces confidence in the observed hierarchy of interventions with respect to outcomes; iv) it was not possible to establish a clear hierarchy among the different therapeutic approaches in terms of combinations, formulations, doses, etc.; v) it was not possible to investigate the impact of the design of the included studies (parallel mouth vs. split mouth) on the primary outcomes due to the insufficient number of trials with both designs for the different comparisons; vi) the small number of studies in each comparison also resulted in low statistical power to detect any possible statistical inconsistencies for PPD and BoP reductions or CAL gain. The same was true for PI, GI, and BI values; vii) finally, the lack of information on adverse effects in most studies prevented their analysis.

## 6 Conclusions and future research

Implications for clinical practice:

(a) Overall, OLO/OzOLO treatments result in a decrease in BoP in the medium term and an improvement in gingival parameters.
(b) Combined therapies appeared to be more effective than single therapy (SRP), but no clear hierarchy could be established.

Future research:

Given the limitations presented, large-scale randomized controlled trials with comprehensive and detailed reports on relevant clinical and radiographic data will be necessary in the future. In addition, future studies should investigate the cost-benefit ratio of combination therapy vs. conventional single therapy.

## Data Availability

The original contributions presented in the study are included in the article/Supplementary Material, further inquiries can be directed to the corresponding author.
