# Supplementary material for: Systematic review and meta-analysis on the effect of olive oil in the treatment of periodontal diseases
Source: Front Oral Health. 2025 Dec 18;6:1735845. doi: 10.3389/froh.2025.1735845 (PMC12756168; doi:10.3389/froh.2025.1735845)
Supplement: Supplementary file 1 [file Table1.docx]

| **Section and Topic** | **Item #** | **Checklist item** | **Location where item is reported** |
| --- | --- | --- | --- |
| **TITLE** | | |  |
| Title | 1 | Systematic review and meta-analysis on the effect of olive oil in the treatment of periodontal diseases. | 1 |
| **ABSTRACT** | | |  |
| Abstract | 2 | Background/Objectives: Periodontal diseases, which are highly inflammatory in nature, are very prevalent worldwide. In recent years, natural products have gained particular attention as a complement to conventional therapy, and olive oil/Ozonated olive oil (OLO/OzOLO), due to its anti-inflammatory and antimicrobial properties, has been proposed for these treatments. The objective of our study was to demonstrate its short-term clinical efficacy in periodontal treatment. Methods: This systematic review and me-ta-analysis was conducted in accordance with Cochrane guidelines, and searches were performed in PubMed, Embase, Cochrane Central, Scopus, and Web of Science (WOS) to identify eligible studies. Review Manager 5.4.1 and SPSS Statistics 30.0® were used to calculate standardized mean differences (SMDs) and 95% confidence intervals (CIs). The main outcomes assessed for periodontitis were probing depth (PDD), bleeding on probing (BoP), and clinical attachment level (CAL), and for gingivitis, plaque index (PI), gingival index (GI) and bleeding index (BI). Results: Twelve randomized clinical trials (RCTs) involving 456 subjects were included. OLO/OzOLO compared to controls improved short-term CAL gain (8–12 weeks) (-0.66; 95% CI [-1.07 to -0.26]; p = 0.001) and reported no benefits in terms of PPD and BoP reduction. In gingivitis, the effect of OLO/OzOLO compared with controls produced a significant reduction in all three indices after 2-8 weeks (-1.52, 95% CI [-2.60 to -0.44]; p = 0.006). Conclusions: Despite limitations, OLO/OzOLO treatments result in short-term CAL gain and improved gingival parameters. | 1 |
| **INTRODUCTION** | | |  |
| Rationale | 3 | Periodontal diseases, which are highly inflammatory in nature, are very prevalent worldwide. In recent years, natural products have gained particular attention as a complement to conventional therapy, and olive oil, due to its anti-inflammatory and antimicrobial properties, has been proposed for these treatments. | 1-2 |
| Objectives | 4 | The objective of our study was to demonstrate the short-term clinical efficacy of olive oil in periodontal treatment. | 2 |
| **METHODS** | | |  |
| Eligibility criteria | 5 | The original research studies were selected according to the following inclusion criteria: (i) randomized clinical trials (single or double blind) with more than 10 participants (n ≥) aged 18 years or over; (ii) that treated periodontal diseases; (iii) that provided data on clinical parameters indicative of periodontal disease; (iv) that used statistical methods, including means and standard deviations, together with units of measurement of mediator levels; (v) without language restriction. Studies that did not meet all the criteria, lacked data on periodontal disease, were experimental studies in animals or in vitro, clinical cases or case series with fewer than 10 patients, literature reviews, and irrelevant studies (editorials, conference contributions, historical reviews, etc.) were excluded. | 4 |
| Information sources | 6 | Table 2 | 4-5 |
| Search strategy | 7 | Table 2 | 5 |
| Selection process | 8 | Table 2 | 5 |
| Data collection process | 9 | Two reviewers (NL-V and AL-V) extracted and tabulated the data from each included study using the standardized data extraction tool “The Joanna Briggs Institute Meta-Analysis of Statistics Assessment and Review Instrument” (JBI-MAStARI). They then reviewed the titles and abstracts of the preselected studies. Those that met the inclusion criteria were read and analyzed in full, and the data they provided were extracted. Discrepancies between reviewers were resolved through discussion. Cohen's kappa (κ) index was used to assess agreement between assessors. Data extracted from the studies included specific details of the populations, methods, specific objectives, and results relevant to the question of interest. They were double tabulated (one tabulation per reviewer) to minimize error bias. | 5 |
| Data items | 10a | List and define all outcomes for which data were sought. Specify whether all results that were compatible with each outcome domain in each study were sought (e.g. for all measures, time points, analyses), and if not, the methods used to decide which results to collect. | ---- |
|  | 10b | List and define all other variables for which data were sought (e.g. participant and intervention characteristics, funding sources). Describe any assumptions made about any missing or unclear information. | ---- |
| Study risk of bias assessment | 11 | the Cochrane Risk of Bias Tool (RoB2, version of August 22, 2019) | 6 |
| Effect measures | 12 | Specify for each outcome the effect measure(s) (e.g. risk ratio, mean difference) used in the synthesis or presentation of results. | ---- |
| Synthesis methods | 13a | Describe the processes used to decide which studies were eligible for each synthesis (e.g. tabulating the study intervention characteristics and comparing against the planned groups for each synthesis (item #5)). | ---- |
|  | 13b | Describe any methods required to prepare the data for presentation or synthesis, such as handling of missing summary statistics, or data conversions. | ---- |
|  | 13c | Describe any methods used to tabulate or visually display results of individual studies and syntheses. | ---- |
|  | 13d | Describe any methods used to synthesize results and provide a rationale for the choice(s). If meta-analysis was performed, describe the model(s), method(s) to identify the presence and extent of statistical heterogeneity, and software package(s) used. | ---- |
|  | 13e | Describe any methods used to explore possible causes of heterogeneity among study results (e.g. subgroup analysis, meta-regression). | ---- |
|  | 13f | Describe any sensitivity analyses conducted to assess robustness of the synthesized results. | ---- |
| Reporting bias assessment | 14 | Describe any methods used to assess risk of bias due to missing results in a synthesis (arising from reporting biases). | ---- |
| Certainty assessment | 15 | Describe any methods used to assess certainty (or confidence) in the body of evidence for an outcome. | ---- |
| **RESULTS** | | |  |
| Study selection | 16a | A total of 726 studies were identified in the databases. After removing duplicates, 355 studies were eligible for evaluation. A total of 336 studies were excluded during the title and abstract screening process. These studies were case series, did not have adequate follow-up, were preclinical studies, were literature reviews, or reported other outcomes. Nineteen full publications were assessed for eligibility. After completing the eligibility assessment, eight studies were excluded (Table 3), and 12 studies were included in this review. | 6 |
|  | 16b | Table 3 | 6 |
| Study characteristics | 17 | Tables 4 and 5 | 7-15 |
| Risk of bias in studies | 18 |  | 18-19 |
| Results of individual studies | 19 | For all outcomes, present, for each study: (a) summary statistics for each group (where appropriate) and (b) an effect estimate and its precision (e.g. confidence/credible interval), ideally using structured tables or plots. | ---- |
| Results of syntheses | 20a | For each synthesis, briefly summarise the characteristics and risk of bias among contributing studies. | ---- |
|  | 20b | Present results of all statistical syntheses conducted. If meta-analysis was done, present for each the summary estimate and its precision (e.g. confidence/credible interval) and measures of statistical heterogeneity. If comparing groups, describe the direction of the effect. | ---- |
|  | 20c | Present results of all investigations of possible causes of heterogeneity among study results. | ---- |
|  | 20d | Present results of all sensitivity analyses conducted to assess the robustness of the synthesized results. | ---- |
| Reporting biases | 21 | Present assessments of risk of bias due to missing results (arising from reporting biases) for each synthesis assessed. | ---- |
| Certainty of evidence | 22 | Present assessments of certainty (or confidence) in the body of evidence for each outcome assessed. | ---- |
| **DISCUSSION** | | |  |
| Discussion | 23a | All studies included in our meta-analysis that evaluated periodontitis used OzOLO. Our meta-analysis did not find a significant reduction in PPD, either in the short term (3-4 weeks) or in the long term (8-12 weeks) of product use. These results would partly agree with those shown by Shoukheba and Ali, who, in a study with 30 patients with localized periodontitis, observed an improvement in periodontal parameters in the group treated with OzOLO after 1 month, but this gradually decreased at 3 and 6 months. Thomé et al., in a systematic review, included studies with all combinations of ozone (gas, water, or oil) and also reported very limited additional benefits in terms of PDD reduction. However, there are clear discrepancies in the literature regarding the benefits of ozone in the complementary treatment of periodontitis. While some studies have found no reduction in clinical parameters when ozone was used as a complement to SRP [55], a recent meta-analysis highlighted its positive effects in com-bination with SRP in terms of PPD and GI indices in patients with periodontitis. We also found no reduction in BoP values, either in the short follow-up periods (3-4 weeks) (p = 0.25) or in the longer periods (8-12 weeks), where only the study by Patel et al. provided data, which were therefore not comparable. These results would be inconsistent with those shown by Grassi et al. (excluded from our meta-analysis because the data were not analyzable), who, in an RCT, evaluated the effects of OzOLO on periodontal pockets (stage II-IV periodontitis) and found significant reductions in BoP at 12 weeks (p < 0.05). Other clinical studies also found no significant improvement in clinical parameters when using ozone combinations associated with SRP [58,59].  The increase in clinical attachment level (CAL) is important, especially because it is the only method for clinically assessing the stability or progression of periodontal disease. Furthermore, it can be easily monitored over time, and in our meta-analysis, we found significant overall values in CAL gain in the intervention groups compared to controls at longer follow-ups (8-12 weeks) (p = 0.001). However, several studies have reported that part of the CAL gain is often lost over time, usually due to patient noncompliance with support programs. | 20-22 |
|  | 23b | In perspective, the present study is limited by the relatively low number of studies available and the amount/type of information collected in them. Consequently, networks for both PPD and BoP reduction, and CAL gain were scarce, due to the low number of direct comparisons and the low number of associated studies, and some of the comparisons were not possible because they were based on a single trial. Therefore, estimates for most comparisons were quite imprecise, which in turn reduces confidence in the observed hierarchy of interventions with respect to outcomes. It was also not possible to establish a clear hierarchical ranking of the different therapeutic approaches, for example, which of the combination treatments is most appropriate in terms of formulations, dosage, etc. It was not possible to investigate the impact of the design of the included studies (parallel versus split mouth) on the primary outcomes due to the insufficient number of trials with both designs for the different comparisons. The small number of studies in each comparison also resulted in low statistical power to detect any possible statistical inconsistencies for PPD and BoP reductions or CAL gain. The same was true for PI, GI, and BI values. Finally, the lack of information on adverse effects in most studies prevented their analysis. | 23 |
|  | 23c | Discuss any limitations of the review processes used. | ---- |
|  | 23d | Discuss implications of the results for practice, policy, and future research. | ---- |
| **OTHER INFORMATION** | | |  |
| Registration and protocol | 24a | The protocol of this meta-analysis has been registered in INPLASY2025100065 (doi: 10.37766/inplasy2025.10.0065). | 3 |
|  | 24b | Indicate where the review protocol can be accessed, or state that a protocol was not prepared. | ---- |
|  | 24c | Describe and explain any amendments to information provided at registration or in the protocol. | ---- |
| Support | 25 | Non-financial support for the review | 22 |
| Competing interests | 26 | The authors declare no conflicts of interest | 22 |
| Availability of data, code and other materials | 27 | Report which of the following are publicly available and where they can be found: template data collection forms; data extracted from included studies; data used for all analyses; analytic code; any other materials used in the review. | ---- |

*From:*  Page MJ, McKenzie JE, Bossuyt PM, Boutron I, Hoffmann TC, Mulrow CD, et al. The PRISMA 2020 statement: an updated guideline for reporting systematic reviews. BMJ 2021;372:n71. doi: 10.1136/bmj.n71
